# Interaction of the Coffee Diterpenes Cafestol and 16-*O*-Methyl-Cafestol Palmitates with Serum Albumins

**DOI:** 10.3390/ijms21051823

**Published:** 2020-03-06

**Authors:** Federico Berti, Luciano Navarini, Elena Guercia, Ana Oreški, Alessandra Gasparini, Jeremy Scoltock, Cristina Forzato

**Affiliations:** 1 Dipartimento di Scienze Chimiche e Farmaceutiche, Università degli Studi di Trieste, via L. Giorgieri 1, 34127 Trieste, Italy; fberti@units.it (F.B.); oreski.ana@gmail.com (A.O.); gasparini.amp@gmail.com (A.G.); jeremyscoltock@gmail.com (J.S.); 2 illycaffè S.p.A., via Flavia 110, 34147 Trieste, Italy; Luciano.Navarini@illy.com (L.N.); Elena.Guercia@illy.com (E.G.)

**Keywords:** serum albumin, diterpenes, coffee, fluorescence, circular dichroism

## Abstract

The main coffee diterpenes cafestol, kahweol, and 16-*O*-methylcafestol, present in the bean lipid fraction, are mostly esterified with fatty acids. They are believed to induce dyslipidaemia and hypercholesterolemia when taken with certain types of coffee brews. The study of their binding to serum albumins could help explain their interactions with biologically active xenobiotics. We investigated the interactions occurring between cafestol and 16-*O*-methylcafestol palmitates with Bovine Serum Albumin (BSA), Human Serum Albumin (HSA), and Fatty Free Human Serum Albumin (ffHSA) by means of circular dichroism and fluorimetry. Circular Dichroism (CD) revealed a slight change (up to 3%) in the secondary structure of fatty-free human albumin in the presence of the diterpene esters, suggesting that the aliphatic chain of the palmitate partly occupies one of the fatty acid sites of the protein. A warfarin displacement experiment was performed to identify the binding site, which is probably close but not coincident with Sudlow site I, as the affinity for warfarin is enhanced. Fluorescence quenching titrations revealed a complex behaviour, with Stern–Volmer constants in the order of 10^3^–10^4^ Lmol^−1^. A model of the HSA-warfarin-cafestol palmitate complex was obtained by docking, and the most favourable solution was found with the terpene palmitate chain inside the FA4 fatty acid site and the cafestol moiety fronting warfarin at the interface with site I.

## 1. Introduction

**Figure 1:**
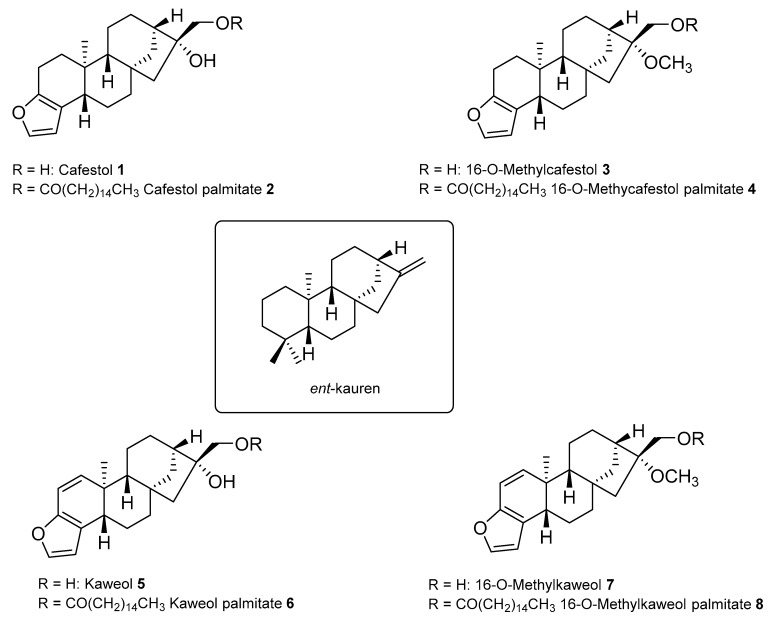
Structure of diterpenes and diterpene palmitates

Today, coffee represents one of the most traded food commodities and its production is remarkably important for the economy of many Countries in South and Central America, Asia and Africa. The coffee plant belongs to the Rubiaceae family, genus *Coffea* which counts more than 100 different species, but the two commercially exploited species are *Coffea arabica*, simply known as Arabica, and *Coffea canephora*, known as Robusta. According to the International Coffee Organization (ICO, 2019) these two species correspond respectively to 57% and 43% of the world’s coffee production and are very different due to genetics, agronomical performance, organoleptic properties and chemical composition. In the coffee seed, the lipid component known as coffee oil is stored in the endosperm tissue as an energy reserve for germination and post-germination growth. The lipid fraction is of particular interest for coffee authentication since it is a powerful chemical tool to differentiate between coffee species and varieties. In this regard, the two main commercially traded coffee species show both quantitative and qualitative differences. From a quantitative point of view, Arabica and Robusta contain between 7% and 17% of coffee oil, the average lipid content of Arabica being significantly higher (15%) than that of Robusta (10%). As far as qualitative aspects are concerned, five lipids classes were identified in green coffee oil: triacylglycerols 75%, unsaponifiable matter 15–18%, partial acylglycerols 5%, free fatty acids 0.5–4.2%, and waxes 1.5–2.5%. About the triacylglycerols portion, the fatty acid profile is similar to that of common edible vegetable oils, with the presence of palmitic, stearic, oleic, linoleic and linolenic acids, being palmitic and linoleic acids the most abundant. The unsaponifiable fraction in coffee oil contains three types of compounds, namely diterpene alcohols, sterols, and tocopherols, whose proportion depends on coffee species, and differences between Arabica and Robusta have been reported [1]. Diterpenes represent the 86–88% of the total unsaponifiable matter for Arabica and approximately 69 % for Robusta, which shows the highest sterolic fraction content (35% of unsaponifiable matter as compared to 10% of Arabica). The diterpenes fraction contains specific pentacyclic alcohols with an *ent*-kauren skeleton that cannot be found in any other food. The major representatives are cafestol **1**, 16-*O*-methylcafestol (16OMC) **3**, kahweol **5**, and the less abundant 16-*O*–methylkahweol **7**. Their profile represents a sort of fingerprint for the identification of geographical and botanical origin as well as for coffee blend’s authentication. (Figure 1)

In Arabica green coffee, cafestol and kahweol are the main components of the diterpenic fraction, as well as of the whole unsaponifiable matter, with an average content of 45%. These two diterpenes are sensitive to acids, light and heat, and kahweol is unstable in its purified form. The unsaponifiable matter from Robusta presents cafestol and 16-*O*-methylcafestol as the two main components but the latter is stable during the roasting process. 16-*O*-methylcafestol has been considered a good chemical marker for detecting Robusta in coffee blends [2] for a long time until than it was detected also in Arabica roasted coffee [3]. However, the presence in Arabica of 16-*O*-methylcafestol in trace amounts makes this compound still a very useful chemical marker for authentication purposes. The diterpenes are mainly esterified with various fatty acids, and only a small amount is found in the free form (less than 3.5%, influenced by storage conditions of green beans). Only a few esters with various fatty acids were reported until 1987 [4], but later on Speer et al. [5] identified several other esters of 16OMC, cafestol and kahweol. Esters of all diterpenes with different fatty acids were identified as well as esters with some odd-numbered fatty acids such as C_17_-C_23_. The odd-numbered fatty acid esters are minor components, whereas the diterpenes esterified with palmitic, linoleic, stearic, oleic, arachidic, and behenic acid exist in larger amounts. The total amount of these six cafestol esters ranges from 9.4 to 21.2 g·kg^−1^ dry weight, corresponding to 5.2–11.8 g·kg^−1^ of cafestol in different Arabica coffees. In Robusta coffees, it was determined to be between 2.2 and 7.6 g·kg^−1^ dry weight, corresponding to 1.2–4.2 g·kg^−1^ of cafestol, less than in Arabica coffees. Although several positive health benefits have been observed for these compounds, such as anti-inflammatory and chemioprotective properties [4], it was also highlighted that they increase the serum cholesterol levels, especially kahweol and cafestol, both in the free form and as palmitate esters [6,7]. Other studies reported anti-angiogenic [8] and anticancer properties [9].

The absorption and excretion process of cafestol has been investigated, and about 70% of the ingested cafestol, administered as its palmitate, is absorbed at the intestinal level, while about 20% is degraded in the stomach environment [10]. In this study, the amount of residual cafestol in the intestinal fluids was evaluated after saponification of the samples, and therefore no data are available on the fraction of palmitate ester that undergoes hydrolysis. This is a general point on the plasma levels of similar natural products, as for instance vegetable sterols, that are present as fatty acid esters but always measured in plasma after saponification. However, there is evidence that steroidal hormones such as estradiol also circulate in plasma as esters and are most likely bound to plasma proteins in this form. In the case of cafestol, the efficiency of protein binding seems vital for understanding the dyslipidemic effects and possible interactions with other bioactive xenobiotics. The competitions that could occur at binding sites on the relative transport proteins can impact on the respective bioavailability and half-lives within circulation [11]. Serum albumin is the main mammalian plasma protein (52–68% of total protein), accounting for over 70–80% of blood osmotic pressure. It is the most relevant representative of plasma transport proteins, endowed with numerous and varied binding sites for endogenous and exogenous molecules, otherwise too poorly soluble for an adequate plasmatic and tissue distribution.

HSA is the most abundant plasma protein with an average physiological concentration of 3–5 g/dL (approximately 600 μM). The transport and regulation functions of these proteins are possible by the numerous binding sites present in their structure; the regulation of site affinity for the ligand can be modulated by pH-dependent conformational microtransitions, competitive displacements and uncompetitive interactions. Of the numerous binding sites, Sudlow I (in the subdomain IIa) and Sudlow II (in the subdomain IIIa) are studied in detail as high-affinity sites (K_A_ ~10^4^–10^6^ L mol^−1^) of interest for the transport of numerous drugs and bioactive molecules [12,13,14]. 

The Sudlow I site, indicated as a binding site for salicylates, warfarin and azapropanone, consists of all 6 helices of the subdomain IIa and a loop of Ib. Its fundamentally apolar pocket houses the FA7 site, probably with higher affinity for medium chain fatty acids [15]. Two polar residues lie towards the bottom of the pocket (Tyr150, His242, Arg257) and at the entrance to it (Lys195, Arg218, Arg222), and the entire cavity is divided into two deep compartments on the right and left through an Ile264 residue, while an anterior compartment is bounded by the aliphatic residues of Leu238, Ala215, Lys199, Arg218 and by the aromatic ones of Phe211 and Trp214 (the only Trp found in the whole protein).

**Figure 2:**
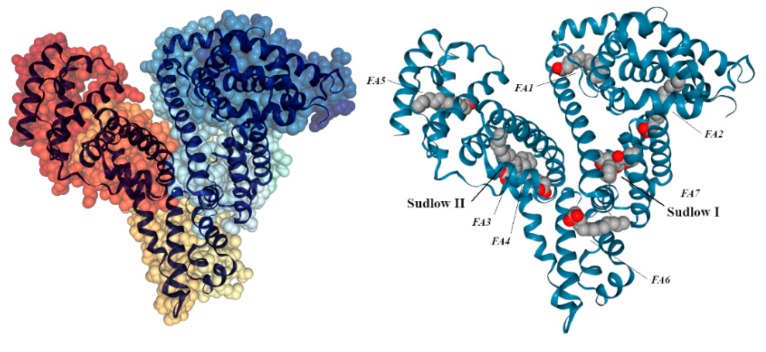
Domains of HSA (left) and map of its binding sites (right).

Studying the warfarin displacement by adding other ligands could lead to important information on the binding site involved in the process. Low concentrations of medium and long chain fatty acids, for example, cause an increase in the affinity of warfarin for HSA (Figure 2).

HSA can be deprived of fatty acids via solvent treatment, giving rise to ffHSA, used in the study of interactions of specific ligands in the presence or absence of fatty acids. BSA is a non-glycosylated globular monomeric protein with a molecular weight of 66 kDa. Its primary structure counts 583 amino acid residues, and the chain is stabilized by 17 disulfide bridges in numerous loops dominated by α-helices. The great structural and functional homology to the HSA, together with the easy accessibility and greater safety of use compared to this, makes it an excellent substitute for the human variant for large-scale use of albumins in research.

We already investigated the binding of serum albumins with cafestol and 16-OMC in free form [16], however, in coffee, diterpenes are mainly esterified with long chain fatty acids. We are now focusing on the interactions of the diterpenes esters with serum albumins to better understand the mechanism of transportation. The structural modifications of the proteins upon the binding event of cafestol palmitate (CP) **2** and 16-O-methylcafestol palmitate (16-OMCP) **4** with HSA, ffHSA and BSA will be examined by circular dichroism spectroscopy. A ligand displacement study with warfarin will be performed for the recognition of the relevant binding site by fluorescence spectroscopy. The same technique will be used to evaluate the equilibrium constants, and finally a model of the HSA-CP complex will be proposed to explain the experimental findings.

## 2. Results and Discussion

The interactions of cafestol palmitate and 16-*O*-methylcafestol palmitate with the three albumins BSA, HSA and ffHSA have been studied first by means of CD spectroscopy, to study changes in the secondary structure of the protein. Our starting hypothesis is that the alkyl chain of the palmitate esters could interact with one of the sites deputed to fatty acids in the proximity of Sudlow I [17]. In our previous work on the free terpenes we have observed binding occurring inside a site very close to the warfarin-binding one actually enhancing its affinity for the protein [15].

### 2.1. Circular Dichroism

**Figure 3:**
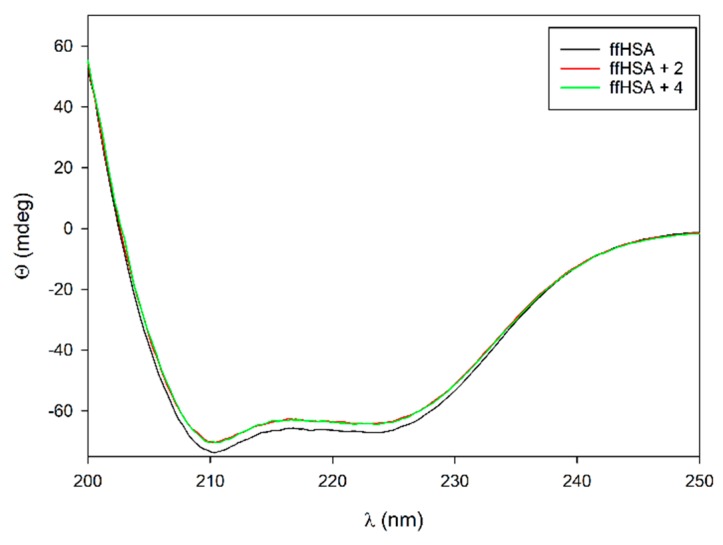
Circular dichroism spectra of 5 μM ffHSA albumin (black) and upon addition of **2** (red) and **4** (green) at 100 µM concentration.

No changes occur in the CD spectra upon the addition of the diterpene palmitates **2** and **4** to the native proteins HSA and BSA up to the maximum concentration of 100 µM. On the contrary, a significant change is observed upon addition of diterpenes palmitates at the concentration of 100 µM to ffHSA, suggesting that in this case the interaction is occurring with a larger change in the secondary structure (Figure 3).

The CD spectra of ffHSA were submitted to deconvolution to evaluate the change in secondary structure. [18] The amount of α-helix decreases to a small extent, from 55.7% to 53.8 and 52.7% in the presence of **4** and **2** respectively. Conversely, the number of parallel structures increases by a similar amount, while the β-turn and coil contents remain unchanged. Although modest, this effect suggests that one of the free fatty acid sites of the protein is involved in the binding, at least when defatted albumin is used. 

### 2.2. Warfarin Displacement Studies

**Figure 4:**
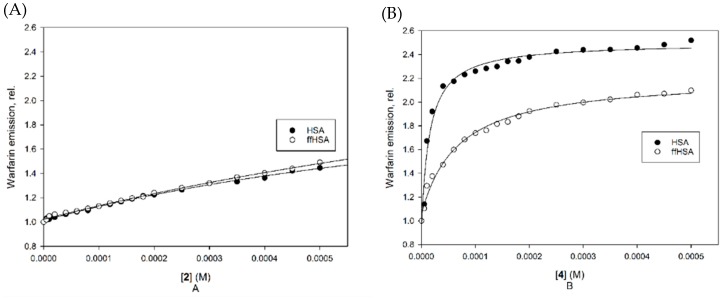
Fluorescence emission of warfarin (excitation 320 nm, emission 380 nm) in the presence of 1 μM albumin and increasing concentrations of **2** (**A**) and **4** (**B**).

A further analysis was carried out on HSA and ffHSA in the presence of warfarin, the reference ligand of Sudlow site I. The intrinsic fluorescence of warfarin, with an emission maximum at 380 nm if excited at 320 nm, is strongly enhanced by its binding to albumin, and it therefore usually decreases when competition with other ligands occurs. A warfarin displacement experiment was thus carried out by adding increasing amounts of **2** and **4** to a preformed albumin-warfarin complex, recording the emission spectrum of warfarin at 25 °C at each addition (Figure 4). In all cases, the emission increases, outlining an enhancement in warfarin affinity following the formation of an HSA-warfarin-ester ternary complex, rather than a competition between ligands. Something similar was already observed in our previous work for the non-esterified diterpenes [15], but in the case of the esters the enhancement is higher. Ligand **4** seems to have a higher affinity for HSA than for ffHSA, as the plateau is reached at lower concentrations with the not-defatted protein. 

This behavior is uncommon among the Sudlow I ligands, which typically compete with warfarin for the binding site, thus suggesting that the diterpenes interact with a binding site adjacent but not coincident with site I. Since in the literature an increase of warfarin affinity was found only in competitive studies with some fatty acids and surfactants, this result reinforces the hypothesis that in binding to ffHSA the palmitate diterpenes occupy a site dedicated to fatty acids, such as FA4, FA6 or FA7, instead of the central part of the Sudlow I pocket. The enhancement of warfarin fluorescence would therefore depend on modifications of the pocket geometry due to the change of secondary structure, or to the rotation of some amino acid side chain. However, the mode of binding to ffHSA appears different from that to the not defatted protein, at least for compound **4**. The differences in affinity were thus further investigated by studying the changes of the protein fluorescence emission. 

### 2.3. Protein Fluorescence

**Figure 5:**
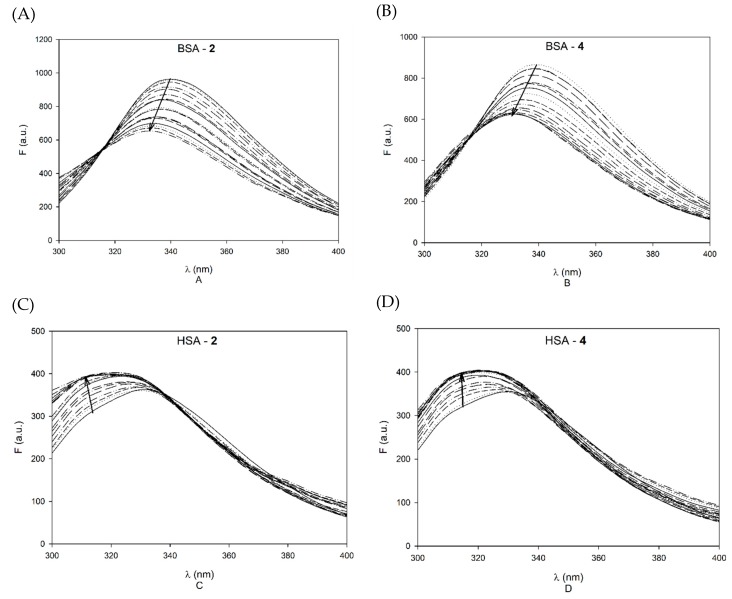

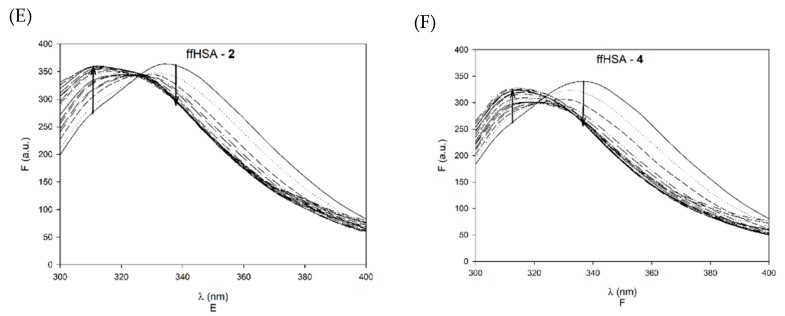
Emission spectra of 1 μM BSA (**A** and **B**), HSA (**C** and **D**) and ffHSA (**E** and **F**) upon addition of increasing amounts of **2** and **4**. The final concentrations of diterpene were 5, 10, 20, 40, 60, 80, 100, 120, 140, 160, 180, 200, 250, 300, 350, 400, 450 and 500 μM.

All the three albumins contain tryptophan, which exhibits fluorescence emission at about 340 nm when excited at 280 nm. HSA and ffHSA have a single tryptophan unit at position 214 while BSA has two tryptophan residues at positions 213 and 134. Trp213 and Trp214 are located inside the Sudlow I hydrophobic pocket. Since the wavelength of the maximum emission of the indole ring (π→π* transition) strictly depends on the micropolarity of the medium and on the capacity of the indole group to form hydrogen bonds, the chemical neighborhood of the residue has a strong impact on its fluorescence emission spectrum. Increasing the polarity of the solvent, the maximum of emission undergoes a shift towards lower energies, or longer wavelengths. Even small variations in the local polarity are able to influence the fluorescence of tryptophan. To study the binding event, fluorescence emission measurements of the albumin tryptophan (280 nm excitation, 300–400 nm emission) were performed upon titration with increasing concentrations of palmitate diterpenes in phosphate buffer at the addition of **2** or **4** aliquots from DMSO mother solutions (at final % of DMSO that do not affect the emission spectra [15,19]), in a range of final diterpene palmitate concentrations of 0–500 µM (Figure 5).

The titrations of BSA emission show the simplest behaviour, as fluorescence quenching is observed, with a blue shift of the 340 nm maximum of 8 nm, and the occurrence of an isosbestic point at 315 nm. On the contrary, HSA emission, which has a less intense maximum at 330 nm, is enhanced rather than quenched on titrations, and again the maximum is blue shifted by over 10 nm. A further different behaviour is observed with ffHSA, where the initial maximum is at 340 nm as in BSA: in this case quenching at 340 nm is coupled with a simultaneous enhancement at 310 nm, and the overall blue shift is remarkably of 30 nm. The occurrence of blue shifts suggests the transition of the fluorophores to a less polar environment, and this is consistent with a reduction of their solvent-exposed surface on binding of the hydrophobic terpene esters. Actually, the initial maximum of not-defatted human albumin is already shifted in comparison to ffHSA due to the presence of fatty acids in binding sites close to the emitting tryptophan, and the hydrophobicity appears to be further improved by the diterpenes. In ffHSA, the effect is very large, most likely due to shielding from water at both the fatty acid binding sites and at further interaction sites. BSA is not defatted, and its emission spectra cannot be compared directly with those of human albumin due to the occurrence of two tryptophan residues, only one of them being affected by interactions with the diterpenes. 

**Figure 6:**
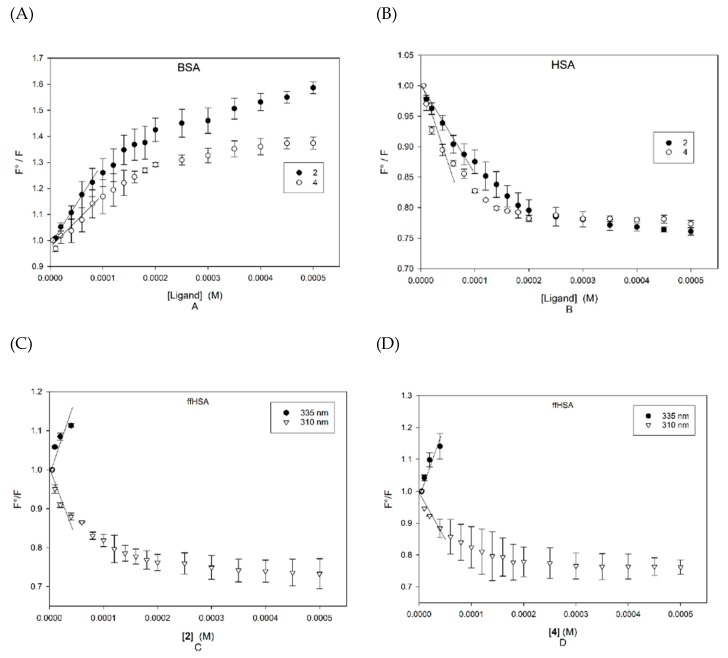
Stern–Volmer plots. (**A**): BSA emission at 340 nm upon addition of **2** (black) and **4** (white). (**B**): HSA emission at 330 nm upon addition of **2** (black) and **4** (white). (**C**): ffHSA emission at 335 (black) and 310 nm (white) upon addition of **2**. (**D**): ffHSA emission at 335 (black) and 310 nm (white) upon addition of **4**.

Despite this complex behaviour, we have carried out a quantitative evaluation of the quenching data. After collecting triplicate measures, and correcting the emission data for slight inner filter effects occurring due to weak absorbance of the diterpenes at 280 nm [20], Stern–Volmer plots were built according to Equation (1):

(1) $$\frac{F_{0}}{F}=1+\;k_{q}\tau_{0}\left[Q\right]=\;1+K_{SV}\left[Q\right]$$

where the amount of quenching is related to the concentration of the quencher, [Q], through the Stern–Volmer constant (K_SV_), that is the product between the kinetic constant of bimolecular quenching (k_q_) and the lifetime of the fluorophore fluorescence in the absence of quencher (τ_0_, in the case of tryptophan about 10^−8^ s [15]). The plots are reported in Figure 6.

The K_SV_ for the two ligands have been obtained by linear regression in the range of low concentrations of ligands, and are reported in Table 1, which also reports the calculated values for k_q_.

Since the kinetic constant k_q_ of the dynamic diffusion-limited quenching at room temperature has an upper limit in the order of magnitude of 10^10^ L/mol s, the major contribution to the fluorescence damping can be regarded as static, at least in the low ligand concentration range (below 100 µM) where K_SV_ have been evaluated. On the contrary, in the higher concentration range, further small effects must be assigned to thermal collisional phenomena. As a general trend, the palmitate diterpenes show less affinity for the three proteins in comparison with the free molecules [16]. The affinities are very similar, and many other small molecules are bound by albumins with similar affinities. Despite the fact that only ffHSA shows significant changes in the CD spectra, the affinity as evaluated in fluorimetry is similar in the defatted and not defatted proteins, and in the bovine protein as well. On the basis of our affinity measures, we can estimate that the fraction of diterpenes bound to albumin after drinking coffee is about 70%. This value was obtained by assuming an average content of 5 mg diterpenes in a cup of espresso coffee, leading to an average maximum concentration in plasma of 6 µM (considering a 70% absorption [10] in a man of 75 Kg weight). As no data are available about the amount of cafestol circulating as ester or free form, an average value of the dissociation constants of the HSA complexes of cafestol and its palmitate has been used. The concentration of albumin has been assumed to be 600 µM. The fluorescence titration experiments show that binding occurs in a region that is close to the emitting tryptophan, and the most intriguing outcome of this study is the evidence that the binding site is not coincident with Sudlow I site, but the interaction of diterpenes activates the classical site, enhancing the affinity for warfarin. This conclusion may be rather relevant, as to the possible interference of coffee terpenes with binding of drugs to albumin, and thus with their pharmacokinetics. Although only 10% of the circulating albumin is bound to cafestol, nevertheless the affinity of such fraction for site I ligands would be greatly enhanced. Since, for instance, warfarin is bound to albumin by 97% and the active fraction is only 3%, the effect could be very high on the absolute concentration of the free drug. In the present study, warfarin was used as the reference compound to map binding at site I. However, it is well known that a wide number of drugs interact with albumin at the same site with similar affinity [13,15].

**Figure 7:**
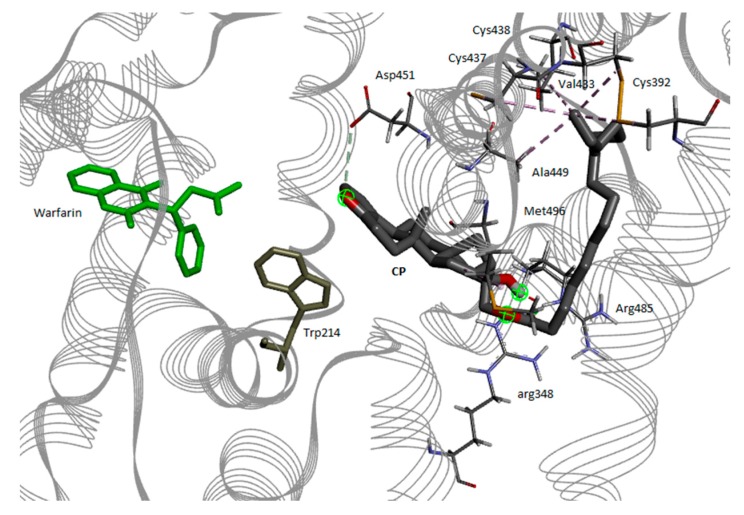
Best docking solution for cafestol palmitate (CP) inside FA4-Sudlow I sites.

Finally, we have carried out a computational analysis with the aim to obtain a model of the HSA-warfarin-**2** ternary complex. In principle, three fatty acid binding sites could be involved in binding. As well as the FA7 site, which occupies the lateral cavity of the Sudlow I pocket, also the FA4 and FA6 sites are closely related to the cavity, sharing one of the lateral and the basolateral side respectively. The myristic acid inside the FA6 site engages Tyr 150, which can rotate towards the polar carboxylic group of the fatty acid to stabilize its interaction with the protein through a hydrogen bond; on the contrary, in the absence of fatty acids, Tyr 150 is directed towards the binding site for drugs within the Sudlow I cavity. The presence of FA7 seems equally important in order to motivate the interaction between fatty acids and ligands in the binding with albumins, due to the sharing of pocket volumes between site I and FA7. As to FA4, Trp 214 itself lies at the interface between the fatty acid head site binding region and the warfarin binding site, and further amino acids such as Asp 451 and Cys 448 are at the borderline. Due to the evidence that ffHSA can bind simultaneously both warfarin and the palmitate esters, we have decided to use as the starting structure that of the warfarin complex with HSA. The coordinate file of the crystal structure of the complex was downloaded from the protein data bank (id. 1H9Z) [17]. This structure contains also all the myristate molecules inside the fatty acid binding sites, besides warfarin. For the setup of the starting geometries, all the myristate molecules were removed from the protein, but warfarin was left inside its binding site. The geometry of the free cafestol palmitate was optimized with the Amber force field, and further conformers besides the one bearing the fully extended alkyl chain were generated with conformations resembling these of the alkyl chain of myristate as found in FA4, FA6 and FA7. Such structures were submitted to docking by Autodock Vina inside the corresponding binding sites. The resulting solutions were then submitted to molecular dynamic annealing and final optimization. All the calculations were carried out with the Amber force field as implemented in Schrödinger 18. The most stable structure was obtained from one of the docking solutions in FA4 (Figure 7).

In this model structure of the ternary complex, warfarin and **2** are close, and the aromatic furan ring of **2** is fronting the carbonyl group of warfarin. Both the ligands are close to Trp 214, from opposite sites. Three polar residues are interacting with the polar groups of **2**, namely Asp 451 with the furan oxygen, Arg 485, and 348 with the hydroxyl and the ester group. The alkyl chain undergoes hydrophobic interactions with Ala 449, Cys 437, Val 433, Cys 438, Cys 392.

This model is actually very different from that suggested in our previous work on binding of free terpenes, where the best solution was found with cafestol inside FP6. A similar solution is impossible for **2** as there is no space in FP6 to host the whole ester. Nevertheless, this solution takes into account all the observations obtained in the present work. Whether the binding mode is the same for free and esterified cafestol, remains an open question.

## 3. Materials and Methods

### 3.1. Materials

HSA (A1653, 96–99%), HSA essentially fatty acid free (ffHSA) (A3782, 99%), BSA (A3912, 96%) were purchased from Merck KGaA. (Darmstatd, Germany) and used without further purification. Their molecular weights were assumed to be 66.478 Da, 66.478 Da and 66.463 Da respectively. Stock solutions of albumins were prepared in phosphate buffer (10 mM in Na_2_HPO_4_ e 2 mM in KH_2_PO_4_, pH 7.4) at concentration of 5 μM. All stock solutions were kept at 4 °C and then diluted to the required experimental sample concentrations. Cafestol palmitate (CP) and 16-O-methylcafestol palmitate (16-OMCP) were synthetized according to the literature [21]. Cafestol palmitate (CP) and 16-O-methylcafestol palmitate (16-OMCP) stock solutions (1. 5 mM and 2.7 mM) were prepared in methanol for circular dichroism analysis and in DMSO for fluorescence analysis.

### 3.2. Circular Dichroism

All titrations were performed at room temperature on a Jasco J-715 Spectropolarimeter equipped with a 0.1 cm path length quartz cuvette. A wavelength range of 200–250 nm was selected and a scan speed of 50 nm/min was chosen. Cafestol palmitate and 16-OMC palmitate were dissolved in 1 mL of methanol to give a 1.5 mM solution. Titrations were performed by keeping the concentration of albumins (BSA, HSA and ff-HSA) fixed at 5 µM in 500 L of phosphate buffer (phosphate buffer 10 mM in Na_2_HPO_4_ and 2 mM in KH_2_PO_4_ diluted, pH 7.4) for all the measurements. Diterpene concentrations varied from 0 µM to 100 µM by adding aliquots of their stock solutions. After each addition of the ligands, a CD spectrum was recorded.

### 3.3. Warfarin Displacement Studies

The displacement of warfarin was studied with a CARY Eclipse (Varian) spectrofluorimeter equipped with a 0.5 cm path length square quartz cuvette, in phosphate buffer (10 mM in Na_2_HPO_4_ and 2 mM in KH_2_PO_4_, pH 7.4). Warfarin was added to the buffer at a 10 µM final concentration from a 1 mM reference solution in DMSO in a 500 L volume. ffHSA was then added at a 1 µM final concentration and the emission spectrum was recorded upon excitation of bound warfarin at 320 nm. Spectra were registered in the range 330–500 nm. The slit width on the excitation was set to 5 nm, on the emission to 10 nm. Cafestol palmitate and 16-OMC palmitate were then added at increasing concentrations by adding aliquots of their stock solutions in the 0–500 µM range, and the emission spectrum was recorded again at each addition.

### 3.4. Fluorescence Spectroscopy

All steady-state fluorescence spectra were recorded at 25 °C on the same spectrofluorimeter and quartz cuvette as described above. An excitation wavelength of 280 nm was used in all cases, and emission spectra were recorded from 290 nm to 500 nm. Different slit width was used depending on the signal intensity: the slit width on the excitation was set to 5 nm, on the emission to 5 nm for BSA spectra while for human albumins the emission slit was set to 10 nm. Quenching experiments were performed by keeping the concentration of albumins fixed at 1 µM in 500 L of solvent phosphate buffer (10 mM in Na_2_HPO_4_ and 2 mM in KH_2_PO_4_, pH 7.4) for all the measurements; diterpenes concentrations varied from 0 to 500 µM by adding aliquots of their stock solutions. All the analyses were replicated three times.

### 3.5. Modelling

The structure of the warfarin-HSA complex was downloaded from the Protein Data Bank and all the crystallization water molecules were removed before the optimization, with the exception of water 13 and 28. All the hydrogen atoms were then added, and the structure was allowed to relax. The Amber force field was used for all the optimizations as implemented in Shrodinger 19 [22]. The model structure of cafestol palmitate was built and optimized to a first minimum having the acyl chain in the fully extended conformation. Further conformations were manually obtained to best fit the conformations of the myristic acid chains as found inside the albumin fatty acid binding sites. Each conformation was then optimized and submitted to docking inside the protein binding area comprising the warfarin site and the neighbouring fatty acids sites. The crystallization fatty acids were removed from the model before carrying out the docking runs, which were performed with Autodock vina [23]. The better geometries were further relaxed as described above, and then allowed to reach thermal equilibrium at 300 °K by a multistep molecular dynamic run in the NTV ensemble (5000 fs at 100 K and at 200 K and then 40,5000 fs at 300 K). Finally, the structures were globally reoptimized to give the final models.

## 4. Conclusions

In conclusion, in this work we have obtained evidence that coffee diterpenes can bind serum albumins also as fatty acid esters, although with less affinities than those of their free forms. Binding occurs in proximity of Sudlow site I, but not inside the site. A fatty acid binding site is also involved, and modelling suggests FA4 as the most likely one. The model suggests a very unusual binding mode, unprecedented if it was confirmed by further experimental evidences as a crystal structure. As to the biological activity of coffee terpenes, this study shows that they could be transported in the blood flow also as fatty acid esters, and delivered without any previous hydrolysis at their action sites, for example, at the farnesoid X receptor. Moreover, they could induce significant changes in drug binding by albumins.

## Figures and Tables

**Table 1:** Stern–Volmer constant K_SV_ and kinetic constant of bimolecular quenching k_q_ according to Stern–Volmer analysis.

	BSA		HSA		ffHSA	
	K_SV_ (L/mol)	k_q_ (L/mol·s)	K_SV_ (L/mol)	k_q_ (L/mol·s)	K_SV_ (L/mol)	k_q_ (L/mol·s)
**2**	2628 ± 123	2.6 × 10^11^	2218 ± 30	2.2 × 10^11^	5233 ± 13	5.2 × 10^11^
**4**	3020 ± 63	3.0 × 10^11^	4810 ± 238	4.8 × 10^11^	3815 ± 74	3.8 × 10^11^

## Abbreviations

BSA	Bovine Serum Albumin
CD	Circular Dichroism
CP	Cafestol Palmitate
DMSO	Dimethylsulfoxide
HSA	Human Serum Albumine
ffHSA	fatty free Human Serum Albumine
16OMC	16-*O*-methylcafestol
16-OMCP	16-*O*-methylcafestol Palmitate

## References

[1] Pablos F., González A.G., Martín M.J., Valdenebro M.S., León-Camacho M. (1999). Determination of the arabica/robusta composition of roasted coffee according to their sterolic content. Analyst.

[2] Pacetti D., Lucci P., Frega N.G. (2015). Unsaponifiable Matter of Coffee. Coffee in Health and Disease Prevention.

[3] Gunning Y., Defernez M., Watson A.D., Beadman N., Colquhoun I.J., Le Gall G., Philo M., Garwood H., Williamson D., Davis A.P. (2018). 16-O-methylcafestol is present in ground roast Arabica coffees: Implications for authenticity testing. Food Chem..

[4] Folstar P., Macrae R. (1985). Lipids. Coffee Chemistry.

[5] Speer K., Kölling-Speer I. (2006). The lipid fraction of the coffee bean. Braz. J. Plant Physiol..

[6] Aro A., Toumilehto J., Kostiainen E., Uusitalo U., Pietinen P. (1987). Boiled coffee increases serum low density lipoprotein concentration. Metabolism.

[7] Bonita J.S., Mandarano M., Shuta D., Vinson J. (2007). Coffee and cardiovascular disease: In vitro, cellular, animal, and human studies. Pharmacol. Res..

[8] Moeenfard M., Cortez A., Machado V., Costa R., Luis C., Coelho P., Soares R., Alves A., Borges N., Santos A. (2016). Anti-Angiogenic Properties of Cafestol and Kawheol Palmitate Diterpene Esters. J. Cell Biochem..

[9] Lima C.S., Spindola D.G., Bechara A., Garcia D.M., Palmeira-Dos-Santos C., Peixoto-da-Silva J., Erustes A.G., Michelin L.F.G., Pereira G.J.S., Smaili S.S. (2017). Cafestol, a diterpene molecule found in coffee, induces leukemia cell death. Biomed. Pharmacother..

[10] De Roos B., Meyboom S., Kosmeijer-Schuil T.G., Katan M.B. (1998). Absorption and urinary excretion of the coffee diterpenes cafestol and kahweol in healthy ileostomy volunteers. J. Intern. Med..

[11] Van Cruchten S.T.J., de Waart D.R., Kunne C., Hooiveld G.J.E.J., Boekschoten M.V., Katan M.B., Oude Elferink R.P.J., Witkamp R.F. (2010). Absorption, Distribution, and Biliary Excretion of Cafestol, a Potent Cholesterol-Elevating Compound in Unfiltered Coffees, in Mice. Drug Metab. Dispos..

[12] Sudlow G., Birkett D.J., Wade D.N. (1975). Characterization of two specific drug binding sites on human serum albumin. Mol. Pharmacol..

[13] Rimac H., Debeljak Z., Boji M., Miller L. (2017). Displacement of Drugs from Human Serum Albumin: From Molecular Interactions to Clinical Significance. Curr. Med. Chem..

[14] Wang Y., Wang S., Huang M. (2015). Structure and Enzymatic Activities of Human Serum Albumin. Curr. Pharm. Des..

[15] Ghuman J., Zunszain P.A., Petitpas I., Bhattacharya A.A., Otagiri M., Curry S. (2005). Structural basis of the drug binding specificity of human serum albumin. J. Mol. Biol..

[16] Guercia E., Forzato C., Navarini L., Berti F. (2016). Interactions of coffee compounds with serum albumins. Part II: Diterpenes. Food Chem..

[17] Petitpas I., Bhattacharya A.A., Twine S., East M., Curry S. (2001). Crystal structure analysis of warfarin binding to human serum albumin: Anatomy of drug site I. J. Biol. Chem..

[18] Micsonai A., Wien F., Bulyàki E., Kun J., Moussong E., Lee Y., Goto Y., Réfrégiers M., Kardos J. (2018). BeStSel: A web server for accurate protein secondary structure prediction and fold recognition from the circular dichroism spectra. Nucl. Acids Res..

[19] Sinisi V., Forzato C., Cefarin N., Navarini L., Berti F. (2015). Interactions of chlorogenic acids and quinides from coffee with human serum albumins. Food Chem..

[20] Fonin A.V., Sulatskaya A.I., Kuznetsova I.M., Turoverov K. (2014). Fluorescence of Dyes in Solutions with High Absorbance. Inner Filter Effect Correction. PLoS ONE.

[21] Finotello C., Forzato C., Gasparini A., Mammi S., Navarini L., Schievano E. (2017). NMR quantification of 16-O-methylcafestol and kahweol in Coffea canephora var. robusta beans from different geographical origins. Food Control.

[22] (2019). Schrödinger Release 2019-4: Maestro.

[23] Trott O., Olson A.J. (2010). AutoDock Vina: Improving the speed and accuracy of docking with a new scoring function, efficient optimization and multithreading. J. Comp. Chem..

